# Early Amyloid-β Toxicity Disrupts Nervous System Connectivity During Aging in *Caenorhabditis elegans*

**DOI:** 10.64898/2026.07.27.740981

**Published:** 2026-07-29

**Authors:** Dilip Kumar Yadav, Christopher W Connor, Christopher V Gabel

## Abstract

Alzheimer’s disease (AD) is characterized by progressive functional neuronal decline ultimately resulting in severe cognitive impairment. However, how neuron function is altered at the cellular level during the critical early stages of the disease and how disease progression relates to the process of normal neuronal aging are poorly understood. To address these fundamental questions, we performed comprehensive multi-neuron imaging in *Caenorhabditis elegans* ( *C. elegans* ) with pan-neuronal expression of human amyloid β _1-42_ peptide (nAβ), the major plaque forming peptide in early AD progression. Measuring neuron activity, connectivity and system wide dynamics with single cell resolution across the *C. elegans* lifespan, we compare Aβ-associated neuronal dysfunction with that of normal aging. Our experiments revealed that nAβ expressing animals exhibit a unique loss of positively correlated neuron connectivity, premature disruption of system wide dynamics, and reduced overall neuronal activity. These neuronal effects correspond to premature impairments in behavior including mechanosensory response as well as the animal’s ability to navigate its environment ( *i.e* ., chemotaxis and thermotaxis). The effects of nAβ expression are distinct from, and in addition to, the process of normal aging that is characterized by a progressive loss of anti-correlated ( *i.e.* inhibitory) neuronal signaling and slower behavioral decline. An increase in resistance to aldicarb, an acetylcholinesterase inhibitor, as well as downregulation of key one-carbon metabolism (OCM) genes ( *metr-1, sams-1* , involved in choline metabolism, the precursor of acetylcholine synthesis) implicate compromised acetylcholine-mediated excitatory transmission in nAβ expressing worms. Supplementation with OCM metabolites (choline, methionine, cysteine) in nAβ expressing worms improved behavior and helped restore OCM gene expression as well as levels of Ach signaling. Likewise, perturbation of the serine synthesis pathway (SSP) that links glycolysis to OCM, altered Ach signaling and OCM gene expression in nAβ animals. Genetic mutations that directly regulate excitatory/inhibitory balance neuronal signaling ( *unc-2* /CaV2α) also help to reduce nAβ-associated behavioral deficits, while NMDA receptor ( *nmr-1* ) mutants showed neither protective effect nor it directly rescue the loss of positive neuron correlativity unique to nAβ animals. Our results demonstrate the unique effects of nAβ toxicity on neuronal dynamics and connectivity as well as the role of key metabolic pathways within the context of a complete, intact, aging nervous system.

## Full Text Availability

The license terms selected by the author(s) for this preprint version do not permit archiving in PMC. The full text is available from the preprint server.

